# Supplementary material for: Compounds without borders: A mechanism for quantifying complex odors and responses to scent-pollution in bumblebees
Source: PLoS Comput Biol. 2020 Apr 22;16(4):e1007765. doi: 10.1371/journal.pcbi.1007765 (PMC7197864; doi:10.1371/journal.pcbi.1007765)
Supplement: S1 Methods — (DOCX) [file pcbi.1007765.s001.docx]

Supplementary Methods

**Odor stimuli**

We selected three essential oils (*New Directions Aromatics)* to serve as odor-blends for this study: lily of the valley, honeysuckle and juniper berry. Lily of the valley (LoV) has been successfully used in odor learning experiments utilizing the proboscis extension reflex (PER) [1]. The two additional odors were selected to provide a range of structural overlap with LoV: the floral-scent honeysuckle served as an ecologically similar odor; while the vegetative odor of juniper berry was selected as the ‘dissimilar’ odor.  Coniferous vegetation is a pungent background in some bumblebee habitats; thus juniper berry represents an ecologically relevant odor pollutant. Blending these three in varying ratios allowed construction of odor stimuli with varying ranges of odorant composition and distance (Table 1). While these essential oils were selected for ecological relevance, commercial essential oils are not likely perfectly-accurate reproductions of their natural odor counterparts (Edwards et al., *in prep*). However, they are multi-component blends that will generate complex stimulation of the bumblebee olfactory system – likely pushing the system towards configural coding [2].  These selected essential oils (EOs), as well as those from the predictive FMPER experiments, were sampled using Solid-Phase Microextraction (SPME) fibers and their composition analyzed with GCMS. For full details please see supplementary methods.

**Odor sampling and characterization of odor-blends**

Selected essential oils (EOs) were sampled using Solid-Phase Microextraction (SPME) fibers (Supelco 57359-U with PDMS/DVB coating) analyzed with a GC-MS.  Thirty microliters of 1:1000 EO dilutions (in odorless mineral oil) were pipetted into glass sampling-vials fitted with a septum cap. The head-space within a vial was allowed to equilibrate at room temperature for 30 minutes, then the SPME fiber was injected and allowed to adsorb odorants for an additional 60 minutes. Fibers were then analyzed using a Shimadzu GC-QP5050A. Samples were injected into a splitless programmed-temperature injector to a Zebron ZB-5MS (5% phenyl, 95% diethylpolysiloxane) of 30 m x .2 mm i.d. x .25 μm film thickness analytical column from phenonenox. The GC temperature program was:  an initial temp of 50 C, hold for 5 min, ramp at 10 c/min, finish at 320, hold for 15 min. All peaks on the GC spectra higher than 10x noise were identified through analysis of MS-spectra peaks with the connected chemical library[3]; the compound with the highest similarity was chosen and all identified peaks had a similarity rating of 80% or above, with the majority of peaks (79%) having a similarity rating of 90% or higher. Once a putative molecular identity was assigned, peaks could be categorized in terms of functional group and carbon chain length. The normalized % area under identified peaks was calculated in LabSolutions.

**Calculation and analysis of expected distribution for odor discrimination with FMPER**

Given that FMPER did indeed provide a reasonable measure of odor-learning (Fig. 2), the response-distribution of bumblebees tested with an AO vs MO should represent a high-contrast odor discrimination task. We used data from the three AO vs MO experiments in this study, as well as four additional AO vs MO experiments from a separate methods study (Edwards et al. *in prep*) to establish the mean correct, incorrect and no-choice responses made by bumblebees in this simple task (Table S2). Given the large sample size of these pooled data (n=177), the entire dataset of bees tested with a learned associative odor (AO) against mineral oil (MO) was used to calculate the percent C, I, and NC and used as the theoretical distribution in the exact tests of goodness of fit (C=61.6%, I=8.5%, NC=29.9%). The level of uncertainty in this distribution was estimated with a probabilistic model where individual silica bees were assigned a correct, incorrect, or no-choice response based on the relationship between a random number from 0-1 and the expected response distribution, where random numbers from 0-0.61699 were assigned “correct”, from 0.617-0.70199 were assigned “incorrect”, and from 0.702-1.0 were assigned “no choice”. Each model run assigned responses to 177 silica-bees (to match the above sample size), from which a C:IC:NC response distribution was calculated. This process was repeated 100 times, and the resulting standard deviation of the estimates for each response category was calculated: SD_C_=3.9, SD_I_ 1.9, SD_NC_=3.7.

References

1. Riveros AJ, Gronenberg W. Olfactory learning and memory in the bumblebee Bombus occidentalis. Naturwissenschaften. Springer; 2009;96: 851–856. doi:10.1007/s00114-009-0532-y

2. Lei H, Vickers N. Central Processing of Natural Odor Mixtures in Insects. Journal Of Chemical Ecology. 2008;34: 915–927. doi:10.1007/s10886-008-9487-2

3. Harris DC. Quantitative Chemical Analysis. 9 ed. New York: W. H. Freeman and Company; 2016.
