# Supplementary material for: Compounds without borders: A mechanism for quantifying complex odors and responses to scent-pollution in bumblebees
Source: PLoS Comput Biol. 2020 Apr 22;16(4):e1007765. doi: 10.1371/journal.pcbi.1007765 (PMC7197864; doi:10.1371/journal.pcbi.1007765)
Supplement: S1 Table — For each method the angle was calculated with two different datasets included in analysis. The ‘subset’ was the original set of three odorants tested in this manuscript: honeysuckle (HS), juniper berry (JB) and lily of the valley (LoV). The complete dataset (‘All’) also included bergamot and cardamom. Because PCA calculates statistical dimensions based upon the input data, the angle between JB and LoV changes with the addition of new odors. CWB angles are based on constant vectors and do not change with additional odors in the analysis. (DOCX) [file pcbi.1007765.s002.docx]

| **method** | **dataset components** | **angle between JB & LoV** |
| --- | --- | --- |
| PCA | subset (HS, JB, LoV) | 114.4 |
| PCA | All | 109.8 |
| CWB | subset (HS, JB, LoV) | 59.7 |
| CWB | All | 59.7 |
