# Supplementary material for: Compounds without borders: A mechanism for quantifying complex odors and responses to scent-pollution in bumblebees
Source: PLoS Comput Biol. 2020 Apr 22;16(4):e1007765. doi: 10.1371/journal.pcbi.1007765 (PMC7197864; doi:10.1371/journal.pcbi.1007765)
Supplement: S2 Table — (DOCX) [file pcbi.1007765.s003.docx]

| **RAW DATA** |  |  |  |  |
| --- | --- | --- | --- | --- |
| Dataset | correct | incorrect | no response | p value (LLR) (compared to null) |
| LoV vs MO | 22 | 5 | 6 | 0.0006 |
| JB vs MO | 15 | 3 | 3 | 0.002 |
| Crd vs MO | 15 | 4 | 11 | 0.033 |
| PM vs MO | 15 | 0 | 5 | 0.000044 |
| SB vs MO | 16 | 0 | 13 | 0.000000088 |
| LD vs MO | 15 | 0 | 12 | 0.000036 |
| ME vsMO | 11 | 3 | 3 | 0.046 |
| **total bees used across experiments = 177** |  |  |  |  |
| Percentages | 0.616 | 0.085 | 0.299 |  |
