# Supplementary material for: Compounds without borders: A mechanism for quantifying complex odors and responses to scent-pollution in bumblebees
Source: PLoS Comput Biol. 2020 Apr 22;16(4):e1007765. doi: 10.1371/journal.pcbi.1007765 (PMC7197864; doi:10.1371/journal.pcbi.1007765)
Supplement: S3 Table — (DOCX) [file pcbi.1007765.s004.docx]

| **Compound** | **Carbon Chain Length** | **Cyclic Carbon Count** | **Functional Groups** |
| --- | --- | --- | --- |
| Eucalyptol | 6 | 6 | oxabicyclic, methyl, monoterpene |
| ocimene | 8 | 0 | alkene, allylicMethyl |
| farnesol | 12 | 0 | alkene, alcohol, allylicMethyl |
