# Supplementary figures and images for: Compounds without borders: A mechanism for quantifying complex odors and responses to scent-pollution in bumblebees
Source: PLoS Comput Biol. 2020 Apr 22;16(4):e1007765. doi: 10.1371/journal.pcbi.1007765 (PMC7197864; doi:10.1371/journal.pcbi.1007765)

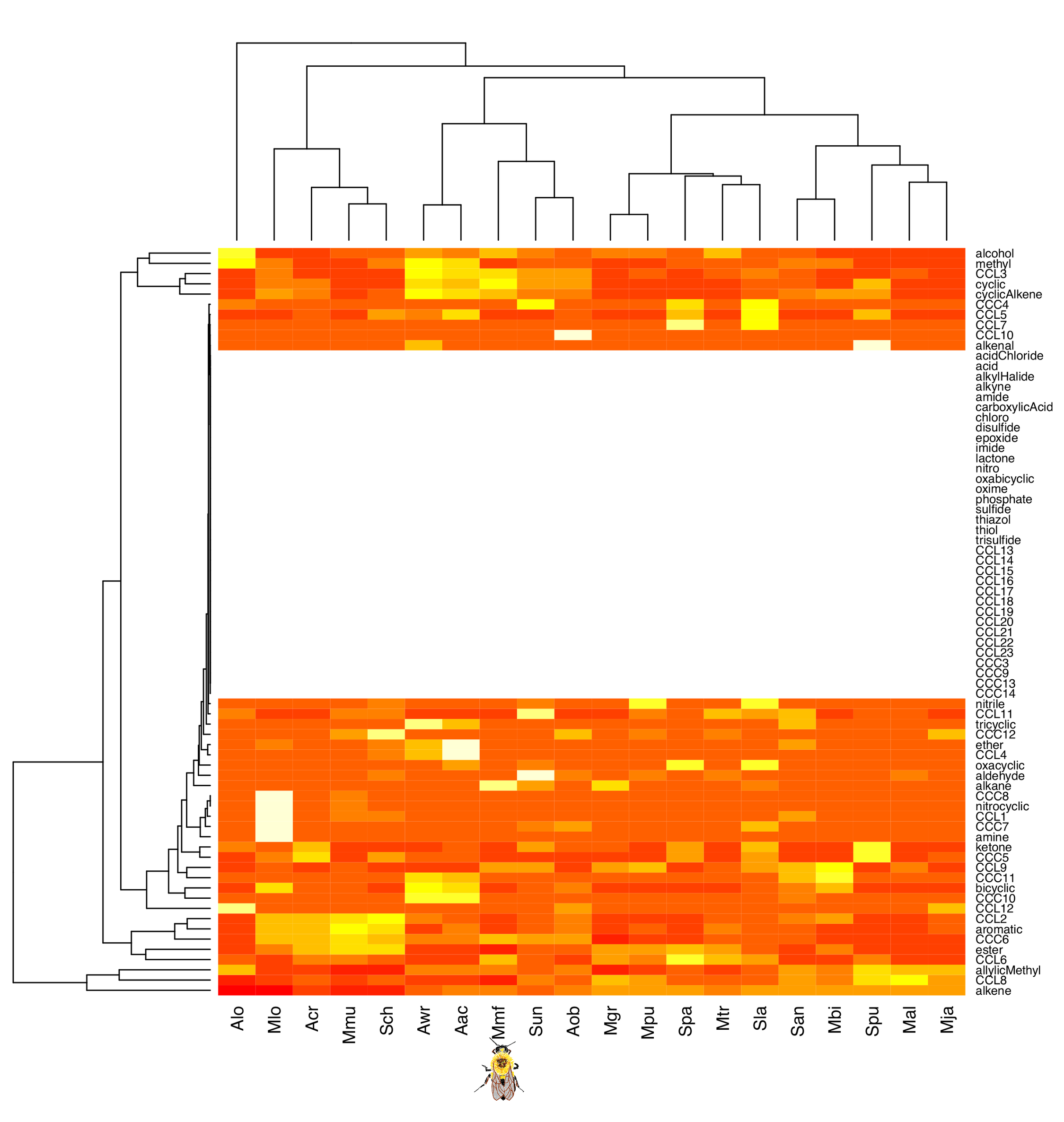

Supplement: S1 Fig — CWB-vector analysis of the twenty different Nyctaginaceae species characterized by Levin et al 2001[4] is shown below. The columns are plant species, while the rows are CWB-dimensions. The heatmap and linkages were created with the heatmap() function in R. The one bumblebee-pollinated species is denoted with a bee. The species key for abbreviations is: Aac = Acleisanthes acutifolia, Acr = A. crassifolia, Alo = A. longiflora, Aob = A. obtuse, Awr = A. wrightii, Mal = Mirabilis alipes, Mbi = M. bigelovii, Mgr = M. greenei, Mja = M. jalapa, Mlo = M. longiflora, Mmf = M. macfarlanei, Mmu = M. multiflora, Mpu = M. pudica, Mtr = M. trifloral, San = Selinocarpus angustifolius, Sch = S. chenopodiodes, Sla = S. lanceolatus, Spa = S. parvifolius, Spu = S. purpusianus, Sun = S. undulates (TIF) [file pcbi.1007765.s006.tif]
