# Supplementary material for: Compounds without borders: A mechanism for quantifying complex odors and responses to scent-pollution in bumblebees
Source: PLoS Comput Biol. 2020 Apr 22;16(4):e1007765. doi: 10.1371/journal.pcbi.1007765 (PMC7197864; doi:10.1371/journal.pcbi.1007765)
Supplement: S1 Appendix — This appendix contains text with all R code necessary to calculate CWB-vectors and angles. (DOCX) [file pcbi.1007765.s007.docx]

Appendix S1: R Code for CWB analysis

#copy to .R file/s and adapt as necessary

##### first section is code to calculate vectors #####

### see the supplemental datasets for how to format your .csv files###

#you will want one for each odor#

#load dimensions from a .csv file with the complete list of dimensions you

#want to consider

setwd(“ ")

dimensions<- read.csv('dimensionMasterList.csv', header=F,stringsAsFactors = FALSE) #see SprayberryDataS1.xlsx for list used in this analysis

unclass(dimensions)

#this has 4 categories of dimensions: functiontional group, total carbon count, carbon chain length, and cyclic carbons

odorantData<- read.csv('compoundDatabase.csv', header=T,stringsAsFactors = FALSE) #see SprayberryDataS1.xlsx for how to format database

# load file with odorant list, should be a .csv with a single column of filenames for #odors

odor_List<-read.csv(file.choose(), header = F,stringsAsFactors = FALSE)

# code to load a .csv file of choice

odorN=dim(odor_List)[1]

#set up vector file for writing data to

vectorfileName<-readline(prompt = "enter vector filename: ")

#start new vector file

setwd(" ")

write.table(dimensions$V1,file=vectorfileName,append=FALSE,row.names = FALSE,col.names="dimensions")

#run a for loop that opens each file on the analysis list, calculates its vector and

#write the data to the vector file

for(g in 1:odorN){

#open up odor composition file

setwd("/Users/jordannasprayberry/Google Drive File Stream/My Drive/Harddrive/Research/CWB Analysis/Odor Blend Structures")

odor_DATAtemp <- read.csv(odor_List[g,1], header = T)

#set up for vector calculations

bob=dim(odor_DATAtemp)

emptyFrame<-data.frame(matrix(nrow=bob[1],ncol=9))

oh = c("area","CCL","CCC","FG1","FG2","FG3","FG4","FG5","FG6")

colnames(emptyFrame)<-oh

# add in area column here

emptyFrame[1:bob[1],1]<-odor_DATAtemp$area

for(i in 1:bob[1]){

idNum=odor_DATAtemp$compoundNumber[i]

emptyFrame[i,2]<-odorantData$CChainLength[idNum]

emptyFrame[i,3]<-odorantData$CCC[idNum]

emptyFrame[i,4]<-odorantData$FG1[idNum]

emptyFrame[i,5]<-odorantData$FG2[idNum]

emptyFrame[i,6]<-odorantData$FG3[idNum]

emptyFrame[i,7]<-odorantData$FG4[idNum]

emptyFrame[i,8]<-odorantData$FG5[idNum]

emptyFrame[i,9]<-odorantData$FG6[idNum]

# print(idNum)

}

odor_DATA<-emptyFrame

#master variable for the calculated dimensions.

vectorCalc = matrix(data=NA,nrow=length(dimensions$V1),ncol=1)

#calculate vector signature

for(i in 1:length(dimensions$V1)){

CCL<-sum(odor_DATA$area[which(odor_DATA$CCL==dimensions$V1[i])])

CCC<-sum(odor_DATA$area[which(odor_DATA$CCC==dimensions$V1[i])])

FG1<-sum(odor_DATA$area[which(odor_DATA$FG1==dimensions$V1[i])])

FG2<-sum(odor_DATA$area[which(odor_DATA$FG2==dimensions$V1[i])])

FG3<-sum(odor_DATA$area[which(odor_DATA$FG3==dimensions$V1[i])])

FG4<-sum(odor_DATA$area[which(odor_DATA$FG4==dimensions$V1[i])])

FG5<-sum(odor_DATA$area[which(odor_DATA$FG5==dimensions$V1[i])])

FG6<-sum(odor_DATA$area[which(odor_DATA$FG6==dimensions$V1[i])])

vectorCalc[i]<-sum(CCL,CCC,FG1,FG2,FG3,FG4,FG5,FG6)

}

#add data to vector file

setwd(" ")

temporary<-read.table(vectorfileName,header=FALSE,sep=",")

vectorData<-c(odor_List[g,1],vectorCalc)

temporary2<-cbind.data.frame(temporary,vectorData)

write.table(temporary2,file=vectorfileName,append=FALSE,sep=",",row.names = FALSE,col.names=FALSE)

}

##### the second section is code to calculate angles #####

#load file

setwd(" ")

d<- read.csv(file.choose(), header=T,stringsAsFactors = FALSE)

attach(d)

focalScent<-readline(prompt = "enter focal vector: ")

focalScentName = focalScent

samples = colnames(d) #list of table headers, will become list for angle #calculations

a=match('dimensions',samples)

samples= samples[-a] #drop dimensions

a=match(focalScent,samples)

samples= samples[-a] #drop focal angle

focalScent=get(focalScent)

d2<-d[,-1] #drop first column of d in d2

rownames(d2)<-d[,1] #use that dropped column as names

attach(d2)

#####

angleData=c(0,0)

# #start angle file -

anglefileName<-readline(prompt = "enter angle filename: ")

write.table(angleData,file=anglefileName,append=FALSE,row.names = FALSE,col.names=FALSE)

# Calculate angle between focal scent and rest in file

#set up for loop to run through the full list of angles

v1=focalScent

for(i in samples){

v2=get(i)

#calculate vector length for all odors

a=v1*v1

vectorLength1 = sqrt(sum(a))

b=v2*v2

vectorLength2 = sqrt(sum(b))

#Dot Products

dotVectors = v1%*%v2

#angle calculation

Theta=acos(dotVectors/(vectorLength1*vectorLength2))

#assemble angle entry

angleName = paste(focalScentName,i,sep="")

angleData<-c(angleName,Theta)

#add to existing vector file

temporary<-read.table(anglefileName,header=FALSE,sep=",")

temporary2<-cbind.data.frame(temporary,angleData)

write.table(temporary2,file=anglefileName,append=FALSE,sep=",",row.names= FALSE,col.names=FALSE)

}
