# Supplementary material for: Compounds without borders: A mechanism for quantifying complex odors and responses to scent-pollution in bumblebees
Source: PLoS Comput Biol. 2020 Apr 22;16(4):e1007765. doi: 10.1371/journal.pcbi.1007765 (PMC7197864; doi:10.1371/journal.pcbi.1007765)
Supplement: Attachment — Submitted filename: Review.docx [file pcbi.1007765.s011.docx]

**Review**

Compounds without borders: a novel paradigm for quantifying complex odors and responses to scent-pollution in bumblebees.

This is an interesting paper that provides a new method of predicting bumblebee odor discrimination behavior using the cosine distance measure of the vectorial representation of the chemicals. The author has shown that this method is capable of replicating the bumblebee behaviour experimentally too, and has identified a range of angles representative of generalizablity and discrimination. It has also been claimed that this method could help in faster screening of odor pollutants in the bumblebee ecology. Although, a similar work has been done in human odor space, wherein Snitz *et al*[1] showed that the odor similarity perceived by humans can be predicted by the cosine distance of the odor structures, a similar result in bees is interesting.

I have a few comments/questions:

1. At the outset, I would to like to request the author to provide page numbers and perhaps line numbers in the manuscript; it helps in pointing out the exact location being referred to.

2. Please define or at least give the full form of the abbreviations before their usage, such as CO (Contrasting odor), Brg- Bergamot and other oils in Table 1. It would make life easier for the readers who are not from a similar background.

3. The title ‘compound without borders (COB)’ although catchy does not seem to justify the context or meaning. Is the author talking about the compounds or the descriptors, or the method which is claimed to be universal in nature? The idea of many to many relationships between compounds and receptors is well known and accepted so, what is so novel about the idea of COB? I think the title should justify the contribution.

4. There are 66 claimed descriptor sets of compounds sufficient to be described as vectors which can be used for distance calculation, how were these specific set and numbers reached at? Please elaborate more on their importance. A simple linear combination of the features is interesting, is there any evidence in this research pointing towards linear relationship of the features governing the neuronal response?

5. What is the meaning of the sentence ‘odor space is constant’? If the odor space is constant, then what is the meaning of different odor in this context?

6. It would be informative to at least name the 5 additional types of associative odor used for comparison task described in Table S2.

7. The calculation of interval sets in identifying the theoretical distribution should be elaborated more, because it forms an important part of the contribution i.e. how and why these ranges were reached at? Moreover, I think the author should also provide the uncertainty involved in reporting expected number of Correct (C), Incorrect (IC) and No Choice (NC), because a point estimation of these values does not convey the real picture. What was the underlying assumption in C, IC and NR i.e. apriori was the assumption that C, IC and NC will be in equal numbers? Was the assumption of posterior a multinomial distribution with the C, IC and NC being independent and P(C) + P(IC) + P(NR) = 1. If yes, then one of the methods could be taking the parameters of the multinomial distribution from a Diritchlet prior.

8. Fig 4 description says subthreshold CO and suprathreshold angles to be 25.1 and 57 respectively but the figure has 24.7 and 56.7, please verify.

9. What is the range of COB angles in bumblebee odor space? Is there a limit to the COB angle in greater than 30 range? Is the 30 degree a hard threshold?

10. The claim of ease of method over PCA seems a bit stretched, because in this method too one has to obtain a 66 dimensional vector first by using GC-MS and then obtain the cosine distance for any inference. PCA is just another statistical step which is to be conducted.

Reference

1. Snitz, K., Yablonka, A., Weiss, T., Frumin, I., Khan, R.M. and Sobel, N., 2013. Predicting odor perceptual similarity from odor structure. *PLoS computational biology*, *9*(9), p.e1003184.
