# Supplementary material for: Compounds without borders: A mechanism for quantifying complex odors and responses to scent-pollution in bumblebees
Source: PLoS Comput Biol. 2020 Apr 22;16(4):e1007765. doi: 10.1371/journal.pcbi.1007765 (PMC7197864; doi:10.1371/journal.pcbi.1007765)
Supplement: Attachment — Submitted filename: SprayberryResponseToReviews.docx [file pcbi.1007765.s012.docx]

**To the editor and reviewers,**

Thank you for your careful and considerate review of this manuscript. I appreciate the reviewers’ time and their positive points such as finding it to be and “interesting finding” and “enthusiasm for the framework”. In reading both reviews it became clear that I had not adequately explained and supported the principle differences between this method for generating dimensions of an olfactory space from statistical methods. As such I have edited the text of the manuscript for clarity and added an explicit comparison of CWB-angles with PCA in the supplemental materials (Table S1). Reviewer 1 expressed concerns about the method used to generate an expected response distribution for statistical tests, which led me to revise my method of calculating the expected response distribution for bumblebee FMPER experiments and update all statistical analyses. Based on Reviewer 2’s observation that the application of CWB-angles to prior studies was inadequate, I have also expanded the analysis of published odor studies with the presented-method. This necessitated rewriting the code for vector and angle calculations to call a central odorant database, and expansion of my existing database of characterized compounds from 140 to just over 360. My responses to each specific reviewer concern is in blue text following the reviewers’ comments below.

I am grateful for the reviewers’ efforts and perspectives, as the manuscript is much improved with the incorporation of these revisions.

Thank you for you reconsideration of this work,

Dr. Jordanna Sprayberry

**Review # 1**

Compounds without borders: a novel paradigm for quantifying complex odors and responses to scent-pollution in bumblebees.

This is an interesting paper that provides a new method of predicting bumblebee odor discrimination behavior using the cosine distance measure of the vectorial representation of the chemicals. The author has shown that this method is capable of replicating the bumblebee behaviour experimentally too, and has identified a range of angles representative of generalizablity and discrimination. It has also been claimed that this method could help in faster screening of odor pollutants in the bumblebee ecology. Although, a similar work has been done in human odor space, wherein Snitz *et al*[1] showed that the odor similarity perceived by humans can be predicted by the cosine distance of the odor structures, a similar result in bees is interesting.

I have a few comments/questions:

1. At the outset, I would to like to request the author to provide page numbers and perhaps line numbers in the manuscript; it helps in pointing out the exact location being referred to.

My apologies for that original oversight, they have been added.

2. Please define or at least give the full form of the abbreviations before their usage, such as CO (Contrasting odor), Brg- Bergamot and other oils in Table 1. It would make life easier for the readers who are not from a similar background.

I have added a key to abbreviations on the final line of Table 1. I also added a brief overview of the behavioral-method to orient readers to the terms associative odor (AO) and contrasting odor (CO) on lines 145-148. Moving through the rest of the manuscript I added full names and a parenthetical reminder of abbreviations when an abbreviation had not been appeared in the text recently (ex: lines 189-190).

3. The title ‘compound without borders (COB)’ although catchy does not seem to justify the context or meaning. Is the author talking about the compounds or the descriptors, or the method which is claimed to be universal in nature? The idea of many to many relationships between compounds and receptors is well known and accepted so, what is so novel about the idea of COB? I think the title should justify the contribution.

The name “compounds without borders” (CWB) does indeed reference the ‘many to many relationships between compounds and receptors” that Reviewer 1 accurately states is a well-supported and accepted phenomenon. The novel aspect of this method is that the mathematical construction of these odor vectors computationally represents those relationships by having multiple compounds with a shared functional group contribute to the power within a single dimension, and to allow a single compound to contribute to multiple dimensions. In this way, an individual compound is not treated as a single entity – it is instead treated as a collection of molecular characteristics. In commonly applied statistical methods, such as principle components or non-metric multidimensional scaling, each unique compound in a dataset represents an input dimension and thus is treated as a discrete entity with its borders intact. Thus the name “compounds without borders”. This ‘CWB” method is indeed similar to the method employed by Snitz et al[1], which certainly does push back against the idea of it being a completely novel method. The principle difference in this study is the selection of broader molecular descriptors based upon the current understanding of olfactory processing pathways in insects, which makes this method accessible to labs without access to expensive molecular databases. The descriptors used by Snitz were provided by proprietary software (Dragon) and statistically optimized to reduce from the original set of 1483 to a set of 21. This reduced set contains far more precise molecular descriptors (ex: Narumi geometric topological index) but they are purely representative of the stimulus molecules themselves, rather than the relationship of those molecules to receptor activation. In this way my work builds upon Snitz et al, rather than recapitulating it in another species. To acknowledge the similarities in the two methods I added a reference to Snitz et al in the methods (lines 431-437). While a catchy name is not necessary, I found it easier to write ‘CWB-angles” rather than ‘angles calculated with the presented method’. If Reviewer 1 would prefer I drop the name and modify the first half of the manuscript title I am more than happy to comply. In addition, I did not intend to communicate that this method is universal in nature – rather that it may have broad applications. How universal this method is will require extensive additional work and could not be determined within the scope of this manuscript. I have edited the applications and limitations section of the manuscript to try and better articulate this for the reader (page 8).

4. There are 66 claimed descriptor sets of compounds sufficient to be described as vectors which can be used for distance calculation, how were these specific set and numbers reached at? Please elaborate more on their importance. A simple linear combination of the features is interesting, is there any evidence in this research pointing towards linear relationship of the features governing the neuronal response?

Regarding dimension selection: The dimensions used in this analysis were determined in collaboration with an organic chemist. Working with Dr. Tim Henry (who did not feel his contributions were significant enough to warrant authorship, which I found mildly disappointing because I would love to coauthor a paper with my father) we looked at the structure of each compound and labelled its functional groups. The starting point for the ‘set’ of functional groups is a standard list that one would find in organic chemistry textbooks. The more detailed/ refined list was generated as Dr. Henry pointed out consistent structures that would impact overall reactivity of molecules. For example, he suggested dividing the methyl dimension into two: methyl and allyic methyls; based on the fact that allylic methyl groups (located on a C with a CC double bond) are more reactive. This process was imprecise in the sense that it evolved from conversations about what reactivity meant in this context – namely that in binding to odorant receptors, volatile molecules are not being reacted into new compounds, but that their general properties likely impact binding probabilities (an idea supported by work such as Hallem et al[2]); however, their success in describing behavior indicates they are reasonable. Future research may allow us to test the viability of this dimensional selection directly, but that is not technology available to me at this time. The included carbon dimensions were chosen based on both the stimulus set and the upper limits to volatility. However, if future odor blends require adding additional carbon dimensions, the presence of those new dimensions will not change existing angle calculations because dimensions with a value of zero do not impact angle values. Thus new dimensions would have been zero in the original data set and would not have caused a shift in the angle-value.

Regarding linearity: The choice to use a linear combination of the relative area under the curve for all relevant component odorants within individual dimensions came from experimental data on lateral neurons (LNs) in the antennal lobe of multiple insect models. The majority of these cells appear to be mediating inhibition within and across glomeruli. The emergent outcome of both this network, as well as aliphatic connections between olfactory receptor is hypothesized to be gain control[3,4]. Given that the predominant gaussian shape of intensity-response curves across sensory systems contains a large linear range, gain control mechanisms should operate to keep the system working in that dynamic range. This means that a linear sum should work when odor concentrations are not too low or too high for the embedded gain control mechanisms to operate. From a practical perspective, without relevant electrophysiology data from bumblebee antennal lobes a linear summation and even weighting of all dimensions was the easiest starting place. However, given that this reasoning is based on the emergent properties of an network that spans the entire antennal-lobe, odors with smaller numbers of components or with constituent molecules that have precise meaning (such as sex pheromones) may not be well described with these angles, as they are channeled through a small subset of glomeruli. I have expanded the discussion (lines 369-381) to better address this limitation of the CWB-method.

5. What is the meaning of the sentence ‘odor space is constant’? If the odor space is constant, then what is the meaning of different odor in this context?

What I hoped to communicate with the phrase ‘odor space is constant’ was the idea that because the axes of the odor space are independent of the data themselves, the relationships between two odors do not change as a dataset expands. Current statistical methods of odor analysis give variable results depending upon the number of odors present in the analysis. I attempted to clarify this idea by editing the text of the introduction (lines 74-82 and 94-104). I have also added a table to the supplemental materials (table S1) that compares the angle between juniper berry and lily of the valley as calculated in principle components space when it includes my original odor set (JB, LoV, and honeysuckle), then when it is derived with my expanded odor set (JB, LoV, HS, cardamom, and bergamot). This shows that the angle between JB and LoV shifts from 114.4° to 109.8° as the data set expands, while the CWB-angle stays constant at 59.7°.

6. It would be informative to at least name the 5 additional types of associative odor used for comparison task described in Table S2.

As requested I have added this to the methods section (lines 476-477).

7. The calculation of interval sets in identifying the theoretical distribution should be elaborated more, because it forms an important part of the contribution i.e. how and why these ranges were reached at? Moreover, I think the author should also provide the uncertainty involved in reporting expected number of Correct (C), Incorrect (IC) and No Choice (NC), because a point estimation of these values does not convey the real picture. What was the underlying assumption in C, IC and NR i.e. apriori was the assumption that C, IC and NC will be in equal numbers? Was the assumption of posterior a multinomial distribution with the C, IC and NC being independent and P(C) + P(IC) + P(NR) = 1. If yes, then one of the methods could be taking the parameters of the multinomial distribution from a Diritchlet prior.

The uncertainty behind the original correct, incorrect, and no choice distribution was not possible to calculate, as I ran the simulation with a large enough number that subsequent model runs returned the same population percentage for C, I, and NC. However, based upon your concerns regarding how I generated the ranges comprising the stochastic model for calculating a theoretical distribution, I consulted a second statistician with more direct expertise in statistical modeling of insect behaviors (Dr. James Russell). His principle concern with my original method was that the large number of silica bees run through the model would over-replicate the system and it would essentially settle on the original means used to generate the ranges. He pointed out the by averaging the **results** of the seven different experiments with different sample sizes I was changing the input weight of some experimental conditions over others (for example AO vs MO results for AO= lily of the valley were measured with 33 bees, while results for AO=juniper berry were calculated from 21 bees; yet the results from each had 1/7 power in the original means). On Dr. Russell’s recommendation I changed the method for calculating a theoretical distribution. Given the large sample size (n=177) I calculated the percent C, I, and NC from the entire dataset of bees tested with a learned associative odor (AO) against mineral oil (MO) and used the result as my expected distribution in the exact tests of goodness of fit (C=61.6%, I=8.5%, NC=29.9%). I then used a probability model to determine the level of uncertainty in this distribution, where individual silica bees are assigned a correct, incorrect, or no-choice response based on the relationship between a random number from 0-1 and the expected response distribution. I ran this model with 177 bees (to match the above sample size) 17 times (to match the number of experiments conducted in this study) to estimate the a standard deviation for each response category (SD_C_=4.1, SD_I_ 2.9, SD_NC_=3.4 ). I edited the manuscript to reflect this updated approach (lines 697-705). The use of a new expected distribution had a minor impact on the results, changing one p-value from 0.09 to 0.04 (AO=juniper berry and CO=3 juniper berry: 1 lily of the valley) and another from 0.04 to 0.09 (AO=juniper berry and CO=1 juniper berry: 1 lily of the valley) . However, given the results of post-hoc binomial comparisons, the change in p-values did not result in a major change in data interpretation.

8. Fig 4 description says subthreshold CO and suprathreshold angles to be 25.1 and 57 respectively but the figure has 24.7 and 56.7, please verify.

In order to implement some of Reviewer 2’s suggestions I modified the code for calculating CWB-angles to operate from a central database of characterized molecules. This resulted in the correction of several minor errors in characterization of cardamom’s and bergamot’s components (the odors from Fig 4). The correct angles are 21.2 for the subthreshold and 45 for the suprathreshold. These have been updated in the text, table 1 and Fig 4.

9. What is the range of COB angles in bumblebee odor space? Is there a limit to the COB angle in greater than 30 range? Is the 30 degree a hard threshold?

In response to Reviewer 2 I expanded the application of CWB-analysis to published studies on olfactory signals in plant-pollinator interactions (page 8). Interspecies angles between different types of floral scents ranged from 6 – 88 degrees; calculated across 5 studies, 58 species, and 56 inter-species comparisons. Only two of these studies directly compared complex odor blend structure with pollinator behavior: one field study on bumblebees and the original lab study on *Manduca sexta* I had characterized. The bee study looked (in part) at the effect of odor manipulation on bee visitation rates. The authors found that adding a novel odorant to two different scent morphs of a single flower species did not change visitation rates. The CWB-angles indicate that these modifications moved the floral odors less than 5 degrees from their original scent structure – a value that is far below the 30 degree threshold measured in this study. While this does not refute our findings, there are far too many differences between the studies for me to feel comfortable claiming that it represents support for a hard 30 degree threshold. The data presented here represent proof of concept and a solidly defined threshold for responses in the FMPER task. However, additional work is required to determine how FMPER thresholds compare to responses in the field. My lab is actively designing methods/ equipment and establishing contacts with local farms in our area to understand the relevance of this work to real work scenarios.

10. The claim of ease of method over PCA seems a bit stretched, because in this method too one has to obtain a 66 dimensional vector first by using GC-MS and then obtain the cosine distance for any inference. PCA is just another statistical step which is to be conducted.

My intent was not to communicate that this method is simpler than PCA. What I hoped to communicate was that the analyzed relationship between two odors stays constant even if the dataset of analyzed odors expands. This constancy should offer better predictive power. I have edited the text of the introduction and discussion to try and better communicate this idea.

Reference

1. Snitz, K., Yablonka, A., Weiss, T., Frumin, I., Khan, R.M. and Sobel, N., 2013. Predicting odor perceptual similarity from odor structure. *PLoS computational biology*, *9*(9), p.e1003184.

Reviewer #2: In MS#COMPBIOL-D-19-01093, by Sprayberry, the author examines an important question in olfactory biology: how do insects process and discriminate complex scents? The study uses a combination of chemical analyses, behavioral (fmPER) and computational methods, the author seeks to examine the probability that an insect can identify different complex scents. I am enthusiastic about the framework of the manuscript, but my enthusiasm is somewhat lessened by the limited scope of the data used in the study.

1. I would appreciate more data analysis in the author's approach. For instance, there has been a tremendous growth in the number of studies showing that floral scents can often cluster based on the primary pollinator 'functional group' (borrowing Fenster et al. 2004 terminology) visiting a floral species. How would using these different datasets change the author's results? Data from Knudsen (1991), Rachel Levin (2001, 2005), Andreas Jurgens and many others could be used to examine whether scent chemistry is predictive of the pollinator group, and how slight changes to the scent alters this link.

The relationship between scent chemistry and pollinator group is a fascinating question, and CWB-vectorization may prove to be a useful tool in investigating them. A thorough exploration of its efficacy would require a careful meta-analysis of the literature, which is outside the prevue of this manuscript. However, I have expanded the analysis of previous literature in the discussion from one study to six studies[5-10] to give readers a better sense of its potential applications. The implications of this expanded analysis are discussed on lines 335-383 of the revised manuscript.

As part of this expansion I modified the code used to calculate CWB vectors and angles to operate from a central database of characterized molecules as the number of considered compounds increased from 140 to 361. This modification of the provided R-code (Appendix S1) should make the CWB-method more accessible for others who wish to test its efficacy in their own research systems.

2. How well does the author's analysis compare to traditional multivariate analyses (NMDS) or machine learning (ML) approaches (random forest)? Although PCA was a traditional analysis early on, more recent methods use ML approaches. Related to the data input, although the author is using the large peaks in the GCMS chromatogram, other smaller peaks can also play an important role in insect perception. This is also reflected in the variation of the scent. The author uses essential oils in the analysis and experiments, but often natural scent can be extremely variable individual by individual. How would this change the author's analysis and results?

In response to the statistical analysis questions: All of the mentioned statistical analyses (PCA, NMDS, and ML) use compound-identity as their input data. The resulting ordination axes of both PCA and NMDS are based upon the input data themselves. Therefore, NMDS is also susceptible to shifts in the statistically derived odor space when the input dataset of odors is modified. This presents similar challenges in terms of predictive abilities. ML is designed to cope with addition of novel data, in that it uses decision trees built on existing data to classify future data. However, it ends with classification rather than a quantitative variable (Larue et al 2015).

In response to the question regarding peak selection from chromatograms: I set the 10x noise threshold for inclusion of peaks in analysis based on the advice of the chemist who trained me on use of the GC-MS in our shared facility on campus, Dr. Christine Ingersol; who recommended using the industry standard for quantitative analysis of 10x noise[11]. I have added this citation to the supplementary methods section.

Finally in response to concerns regarding essential oils, I believe that the essential oils were an effective stimulus in this study because of their complexity. I have edited the discussion section on applications and limitations of CWB-angles to better address the role of stimulus complexity (lines 369-381). That said, I agree that essential oils are not necessarily accurate recreations of natural odors, as stated in the supplemental methods: “While these essential oils were selected for ecological relevance, commercial essential oils are not likely perfectly-accurate reproductions of their natural odor counterparts (Edwards et al., in prep). However, they are multi-component blends that will generate complex stimulation of the bumblebee olfactory system – likely pushing the system towards configural coding [12].” The in prep manuscript referenced here is a methods manuscript on a mechanism for using plant material to develop ecologically relevant odor stimuli for experiments.

3. The author also brings up an important point about how scent pollution may alter these scent profiles. But there is a gap in these hypotheses and the analyses performed. For instance, ozone and NO3 will modify the scent composition, but the author does not address how such degradation may be reflected in the analysis. If the author adds an agrochemical pollutant, how does that modify the scent differential, and influence behavior using FMPER?

I consider scent pollution to be any outside influence that changes the original odor. By this definition scent pollution could happen in both natural and anthropogenic environments. The original focus of my work dealt with additive pollution via agrochemical odors, which influenced my decision to employ additive odor pollution (by blending with other essential oils) in these experiments. I have clarified this in lines 62-67 of the introduction. Now that we have proof of concept with this dataset my lab is starting to move forward with investigating the efficacy of CWB-angles to describe and predict the effects of agrochemical with both field and lab components. We are in the process of working with local farmers to determine which agrochemicals they use, so we know which are the most impactful to include in testing. In addition I am in discussing a potential collaboration with a lab focused on the degradation effects of ozone on floral odors. The negative effects of this would be quantified by calculating a normal vector, a degraded vector, and the resulting angle between them. These are exciting potential applications of this work, but are outside the scope of the current manuscript.

4. Moreover, with respect to the insect, datasets exist for functional characterization of the odor constituents activating the olfactory sensory neurons (Drosophila, Anopheles, Tephritids, others...)(see Biasazin et al., 2018) - can the author use related dataset(s) to match the chemistry with the insect perception?

These types of data sets informed the computational approach to vectorizing odors presented here. I have endeavored to clarify this in the introduction (lines 81-98). I did not explicitly use individual olfactory receptor proteins as the dimensions for CWB vectors for several reasons. One, the relevant datasets for bumblebees do not exist. Two, if the extensive lateral interactions both within the antenna and the antennal lobe[3,4] were not mathematically accounted for in vectorization the resultant angles would likely be more predictive of input to the antennal lobe than of the emergent animal behavior. However, these lateral interactions are likely to have a larger impact on more complex input odor stimuli. Therefore, if the CWB method is effective at describing the emergent properties of olfactory processing of complex odors, it may be less effective at characterizing and predicting responses to more simplistic stimuli. CWB-analysis of the scent stimuli used in figure 4 of Perez et al [10]supports this idea. I have added this analysis to the ‘applications and limitations’ section of the discussion (lines 369-381).

5. The author brings up many different topics in the Introduction and Discussion (scent discrimination and composition, encoding of the scent, scent pollutants, etc... From the readers perspective, it would help to focus the Intro on a few testable hypotheses that relate to the experiments in the Results: what is the relationship between complex scents and insect perception?

I have edited the introduction to try and clarify its narrative. I now explicitly state the goal of the manuscript (lines 79-81) and use the remaining half of the introduction to explain the relationship of my approach to odor dimensionalization to existing literature on the neurophysiology of odor processing in insects. I hesitate to reduce the content included in the introduction given the broad readership of PLoS Computational Biology. Additionally, as a professor at a small liberal arts college I read an extensive amount of literature with undergraduates. I find that papers whose introductions provide background information on the reasoning behind experimental questions are more accessible to young scientists – an observation that impacts the way I now approach my disciplinary writing. However, if the reviewer would prefer that I remove these topics I am happy to comply.

**Have all data underlying the figures and results presented in the manuscript been provided?**
Large-scale datasets should be made available via a public repository as described in the *PLOS Computational Biology* [data availability policy](http://journals.plos.org/ploscompbiol/s/data-availability), and numerical data that underlies graphs or summary statistics should be provided in spreadsheet form as supporting information.

Reviewer #1: Yes

Reviewer #2: No: The author mentions other datasets, but this is not included in the manuscript.

All the odor data are available in the file ‘SprayberryDataS1.xlsx’ and all the behavior data are available in the file ‘SprayberryDataS2.xlsx’

**Literature Cited**

1.	Snitz K, Yablonka A, Weiss T, Frumin I, Khan RM, Sobel N. Predicting Odor Perceptual Similarity from Odor Structure. Diedrichsen J, editor. PLoS Comput Biol. 2013;9: e1003184. doi:10.1371/journal.pcbi.1003184.s004

2.	Hallem EA, Carlson JR. Coding of Odors by a Receptor Repertoire. 2006;125: 143–160. doi:10.1016/j.cell.2006.01.050

3.	Clifford MR, Riffell JA. Mixture and odorant processing in the olfactory systems of insects: a comparative perspective. J Comp Physiol A. 2013;199: 911–928. doi:10.1007/s00359-013-0818-6

4.	Martin JP, Beyerlein A, Dacks AM, Reisenman CE, Riffell JA, Lei H, et al. The neurobiology of insect olfaction: Sensory processing in a comparative context. Progress in Neurobiology. 2011;95: 427–447. doi:10.1016/j.pneurobio.2011.09.007

5.	Levin RA, Raguso RA, McDade LA. Fragrance chemistry and pollinator affinities in Nyctaginaceae. Phytochemistry. 2001;58: 429–440.

6.	Riffell JA, Lei H, Abrell L, Hildebrand JG. Neural Basis of a Pollinator's Buffet: Olfactory Specialization and Learning in Manduca sexta. Science. 2013;339: 200–204. doi:10.1126/science.1225483

7.	Odell E, Raguso RA, Jones KN. Bumblebee foraging responses to variation in floral scent and color in snapdragons (Antirrhinum: Scrophulariaceae). Am Midl Nat. 1999.

8.	Johnson SD, Jürgens A. Convergent evolution of carrion and faecal scent mimicry in fly-pollinated angiosperm flowers and a stinkhorn fungus. South African Journal of Botany. SAAB; 2010;76: 796–807. doi:10.1016/j.sajb.2010.07.012

9.	Jürgens A, Witt T, Gottsberger G. Flower scent composition in Dianthus and Saponaria species (Caryophyllaceae) and its relevance for pollination biology and taxonomy. Biochemical Systematics and Ecology. 2003;31: 345–357. doi:10.1016/S0305-1978(02)00173-4

10.	Perez M, Giurfa M, d'Ettorre P. The scent of mixtures: rules of odour processing in ants. Sci Rep. 2015;5: 628–9. doi:10.1038/srep08659

11.	Harris DC. Quantitative Chemical Analysis. 9 ed. New York: W. H. Freeman and Company; 2016.

12.	Lei H, Vickers N. Central Processing of Natural Odor Mixtures in Insects. Journal Of Chemical Ecology. 2008;34: 915–927. doi:10.1007/s10886-008-9487-2
