# Supplementary material for: Compounds without borders: A mechanism for quantifying complex odors and responses to scent-pollution in bumblebees
Source: PLoS Comput Biol. 2020 Apr 22;16(4):e1007765. doi: 10.1371/journal.pcbi.1007765 (PMC7197864; doi:10.1371/journal.pcbi.1007765)
Supplement: Attachment — Submitted filename: Sprayberry Response to Reviews.docx [file pcbi.1007765.s013.docx]

**To the editor and reviewers,**

**Thank you for your careful and considerate review of this manuscript. I appreciate the reviewers’ time and have responded to all of their comments. My responses are in blue text below each individual reviewer comment. I am grateful for the reviewers’ efforts and perspectives, as the manuscript is much improved with the incorporation of these revisions.**

Reviewer #1: Review
Compounds without borders: a novel paradigm for quantifying complex odors and responses to
scent-pollution in bumblebees.

The line numbers specified here point to the modified new manuscript.
The author has made some specific changes to the manuscript and I have a few comments/suggestions on the new version.

1. In reference to the statements.

‘...then used a probability model to determine the level of uncertainty in this distribution, where individual silica bees are assigned a correct, incorrect, or no-choice response based
on the relationship between a random number from 0-1 and the expected response distribution. I ran this model with 177 bees (to match the above sample size) 17 times (to
match the number of experiments conducted in this study) to estimate the a standard deviation for each response category (SD C =4.1, SD I 2.9, SD NC =3.4 ). I edited the manuscript to reflect this updated approach (lines 697-705).’

I am afraid, the method described here is still not clear, for example which probability model was used to assign correct, incorrect, no-choice response. I understand it has been said to depend upon the relationship between a random number from 0-1 and the expected distribution but, does it depend upon the proportion of the observed distribution? To be exact, what was the apriori assumption in assigning the random number to a particular class of C, IC, NC? I think the usage of terms expected and observed is confusing. The expected in general comes out of the theoretical distribution and observed is what is found from actual experiments.
Moreover, the number of runs to get the SDs are too less (they should be run at least 100 times to get a proper distribution i.e. it is not sufficient to match the number of experiments done practically).

I have edited and expanded the text of this section (lines 709-726 ), which is pasted below:

“Given that FMPER did indeed provide a reasonable measure of odor-learning (Fig. 2), the response-distribution of bumblebees tested with an AO vs MO should represent a high-contrast odor discrimination task. We used data from the three AO vs MO experiments in this study, as well as four additional AO vs MO experiments from a separate methods study (Edwards et al. *in prep*) to establish the correct, incorrect and no-choice responses made by bumblebees in this simple task (Table S2). Given the large sample size of these pooled data (n=177), the entire dataset of bees tested with a learned associative odor (AO) against mineral oil (MO) was used to calculate the percent C, I, and NC and used as the theoretical distribution in the exact tests of goodness of fit (C=61.6%, I=8.5%, NC=29.9%). The level of uncertainty in this distribution was estimated with a probabilistic model where individual silica bees were assigned a correct, incorrect, or no-choice response based on the relationship between a random number from 0-1 and the expected response distribution, where random numbers from 0-0.61699 were assigned “correct”, from 0.617-0.70199 were assigned “incorrect”, and from 0.702-1.0 were assigned “no choice”. Each model run assigned responses to 177 silica-bees (to match the above sample size), from which a C:IC:NC response distribution was calculated. This process was repeated 100 times, and the resulting standard deviation of the estimates for each response category was calculated: SD_C_=3.9, SD_I_ 1.9, SD_NC_=3.7.”

As you can see in this text I increased the iterations of the model to estimate standard deviation to 100. I have also replaced ‘expected’ with ‘theoretical’ as requested. I made similar modifications to the methods text within the main body of the manuscript (line 518), and in the caption for table S2 (line 740). As with the first round of the manuscript, the apriori assumption is that the total probability of all scenarios will sum to 1; this assumption dictated how random numbers were assigned a ‘response’.

2. One more thing that is concerning is the less number of examples of chemicals between 20-29 degrees range (not the samples or replicates). Any claim in this range has to be verified with more data or stated clearly to be inconclusive.

This study represents a represents proof of concept, and I see it as a starting point rather than an ending. I have therefore replaced the definitive language with conditional language in the discussion (lines 310-318). That said, the 20-29 degree range has a similar number of replicates when compared to the other relevant ranges: seven of the odor-discrimination tasks presented in this study directly test how bumblebees respond to a contaminated- versus an uncontaminated-learned odor. Of these seven, two are in the 10-19 degree range, three are in the 20-29 degree range, and two are in the >30 degree range. Future work will test the ecological and ethological relevance of signals in the 10-19 degree range versus the 20-29 degree range.

3. Line 65- ‘Scent contaminants cause an additive-modifiction of scent blends.’.. please correct the spelling of modification.
Does the author have some evidence to back this claim as this is an interesting and strong statement, the change of bumblebee behaviour says nothing about additive/multiplicative or any such modification? It only says there is a change. There has to be some evidence in physico-chemical and neural space to claim this.

What I was endeavoring to communicate here is that changes of behaviorally relevant odors could happen (generally) in two ways: 1) by removing components (subtractive); or 2) by adding components (additively). I reference literature working on subtractive odor pollution in lines 65-67. I have changed the text of the referenced line (‘Scent contaminants cause an additive-modification of scent blends.’) to “Scents can also be contaminated by the addition of novel odors, resulting in a modified of odor-blend.”, lines 67-68, in order to communicate this idea more clearly. I was not trying to imply that individual odorants are interacting in a summative, linear fashion at the level of the olfactory receptor neurons or at the level of the antennal lobe. That conclusion, as you rightly point out, would require direct testing of bumblebee ORNs, hopefully followed by recordings from the antennal lobe. However, as addressed more directly in the response to #4, CWB vectors do not represent a *neural*  space, they represent a *physical* space.

4. Line 79-81, ‘The principle goal of this study was to develop an odor-space with quantitative independent-axes that could be used for both descriptive and predictive analysis of bumblebee behavior’.
If the author claims to have found out independent axes in bumblebee odor space then it has has to be verified mathematically and analytically, what are these axes, how are vectors related in this space, is this a euclidean space? Because this is a long and often elusive question in the odor space research i.e. to find independent axes. I think the main contribution of this paper is to provide a new method of predicting bumblebee odor discrimination behaviour using the cosine distance measure of the vectorial representation of the chemicals. The claim of independent axes in my humble opinion should be avoided.

At first read I was unsure how to respond to this concern because from my perspective the second to last sentence answered the questions posed in the second sentence. Upon further reflection I think I understand where the miscommunication resides. I think that reviewer 1’s interpretation is that I am proposing that CWB vectors are a set of neural rules for how bumblebees process odor. However, CWB vectors are a Euclidean representation of a physical odor space. This is akin to the difference between a reflectance-spectra measuring a flower’s color versus calculating that flower’s location in a honeybee’s color hexagon (Chittka 1992). The reviewer is absolutely correct, these experiments do not support the conclusion that CWB vectors model odor representation within a bumblebee brain. Happily, that is not what I was trying to accomplish. Rather, my goal was to craft a mathematical representation of scent that allows for comparison of complex odors – much as one could compare the reflectance-spectra of two different flowers to quantify their color differences. I consider the dimensions of CWB-vectors to be ‘independent’ because they are independent of the odor stimuli themselves – much as wavelength exists independently of flowers. Current statistical methods of odor analysis create principal component axes that are dependent upon the identity of the molecular constituents of the odors being analyzed. This is why, as demonstrated in Table S1, the relationship between two odors changes in principle component space if the number of odors analyzed increases. To better help readers understand this distinction I have added text (lines 121-123) directly referencing a visual analogy. In addition I have uploaded a supplemental video (S1) that animates figure 1, giving readers a more thorough explanation of how CWB-vectors are constructed.

Chittka L. 1992. The colour hexagon: a chromaticity diagram based on photoreceptor excitations as a generalized representation of colour opponency. J Comp Physiol A. 170: 533-543.

5. I am still not convinced with the term compound without border (CWB) and its use as title. I will let the author decide the relevance of use of this term as title.

Thank you.

6. Line 174- ‘..discriminate between an associative odor (AO) and unscented mineral oil (Fig. 2C)..’
there is no figure 2C. Please verify.

Apologies, 2C in the original manuscript became 2B. This has been fixed (line 202).

7. Line 198- ‘..LoV-associated bumblebees and 55.6% for JB-associated (Fig. 4).’
Is it fig 4 or fig 3 ?

(Now line 227) It is figure 3, thank you.

8. Fig. 5 X-axis should have more markers in X-axis to depict the angles more clearly.

I have added minor gridlines every 4 degrees.

9. Line 242 - ‘.. and a suprathreshold CO ((theta= 57, Crd vs Brg...’
The caption says 57 but the figure has theta = 45. please verify.

The angles in the figure legend have been corrected.

10. Line 351- ‘..in the alkane and cyclic dimensions than any of the other odors (Fig S?).’
Please check which figure is being referred to.

(line 388) Figure S1, apologies.

Reviewer #2: In the revised manuscript, the author carefully addresses the reviewer comments. I think the revised manuscript is almost ready for prime time. I only have a few minor comments that should be easily addressed.

1. Introduction. The Intro, as rendered in the pdf, is difficult to read, perhaps because it is a single, long paragraph, with many distinct topics. For readability, I would encourage the author to separate the Introduction into distinct paragraphs, each with a topic sentence and concluding sentence to provide continuity between sections while addressing the different topics. For example:
A. The importance of floral advertisements and scent as an important cue that acts as a long-distance attractant.
B. How floral scents are subject to contaminants (anthropogenic and natural).
C. Statistical methods do not adequately enable predictive ability to characterize these dynamics.
D. Insect olfactory processing, CWB, and the discrete hypotheses (or questions) addressed in this manuscript.
 I opted for breaking the introduction into topical sections with descriptive headings that largely mirror these suggestions. A reader skimming the bold topic sentences would now get a clear outline of what information is addressed.

2. For clarity and avoiding anthropomorphic terms, I would suggest changing a few phrases in the manuscript:
A. Fig. 1 Legend (Lines 133-135): Change "species" to "type" to avoid confusion about the bee or moth species used in the manuscript.
B. Line 108: Change "mechanism" to "analysis".
C. Change heading title on Line 247 to "Scent contamination reduces odor discrimination". We're not sure that the scent is unrecognizable to the bee (could be "recognizable" depending on context, bioassay, etc..).

2A (now 150), 2B (now line 128), 2C (now line 279) all changed

3. Line 351: Insert the supplemental figure number.
 Done (now line 388)

**Have all data underlying the figures and results presented in the manuscript been provided?**
Large-scale datasets should be made available via a public repository as described in the *PLOS Computational Biology* [data availability policy](http://journals.plos.org/ploscompbiol/s/data-availability), and numerical data that underlies graphs or summary statistics should be provided in spreadsheet form as supporting information.

Reviewer #1: Yes

Reviewer #2: Yes

PLOS authors have the option to publish the peer review history of their article ([what does this mean?](https://journals.plos.org/ploscompbiol/s/editorial-and-peer-review-process#loc-peer-review-history)). If published, this will include your full peer review and any attached files.


If you choose “no”, your identity will remain anonymous but your review may still be made public.


**Do you want your identity to be public for this peer review?** For information about this choice, including consent withdrawal, please see our [Privacy Policy](https://www.plos.org/privacy-policy).

Reviewer #1: No

Reviewer #2: No
